# The analysis of *GSTA1* promoter genetic and functional diversity of human populations

**DOI:** 10.1038/s41598-021-83996-2

**Published:** 2021-03-03

**Authors:** Vid Mlakar, Patricia Huezo-Diaz Curtis, Marc Armengol, Victor Ythier, Isabelle Dupanloup, Khalil Ben Hassine, Laurence Lesne, Rabih Murr, Simona Jurkovic Mlakar, Tiago Nava, Marc Ansari

**Affiliations:** 1 Research Platform for Pediatric Oncology and Hematology, Faculty of Medicine, University of Geneva, Avenue de la Roseraie 64, 1205 Geneva, Switzerland; 2 Department of Genetic Medicine and Development, University of Geneva Medical School, 1211 Geneva, Switzerland; 3 Swiss Institute of Bioinformatics, 1015 Lausanne, Switzerland; 4 Pediatric Oncology and Hematology Unit, Department of Women, Children and Adolescents, Geneva University Hospital, Rue Willy-Donzé 6, 1205 Geneva, Switzerland

**Keywords:** Biomarkers, Molecular medicine, Gene expression, Genetic markers, Haplotypes, Medical genetics, Population genetics

## Abstract

*GSTA1* encodes a member of a family of enzymes that function to add glutathione to target electrophilic compounds, including carcinogens, therapeutic drugs, environmental toxins, and products of oxidative stress. *GSTA1* has several functional SNPs within its promoter region that are responsible for a change in its expression by altering promoter function. This study aims to investigate distributions of *GSTA1* promoter haplotypes across different human populations and to assess their impact on the expression of *GSTA1*. PHASE 2.1.1 was used to infer haplotypes and diplotypes of six *GSTA1* promoter SNPs on 2501 individuals from 26 populations classified by the 1000 Genomes Project into five super-populations that included Africa (N = 660), America (N = 347), East Asia (N = 504), Europe (N = 502), and South Asia (N = 488). We used pairwise FST analysis to compare sub-populations and luciferase reporter assay (LRA) to evaluate the impact of each SNP on activation of transcription and interaction with other SNPs. The distributions of *GSTA1* promoter haplotypes and diplotypes were significantly different among the different human populations. Three new promoter haplotypes were found in the African super-population. LRA demonstrated that SNPs at -52 and -69 has the most impact on *GSTA1* expression, however other SNPs have a significant impact on transcriptional activity. Based on LRA, a new model of cis-elements interaction is presented. Due to the significant differences in *GSTA1* diplotype population frequencies, future pharmacogenomics or disease-related studies would benefit from the inclusion of the complete *GSTA1* promoter haplotype based on the newly proposed metabolic grouping derived from the LRA results.

## Introduction

The glutathione-S-transferases (GST) are a group of enzymes that catalyse the addition of glutathione to target electrophilic compounds, including carcinogens, therapeutic drugs, environmental toxins, and products of oxidative stress^1,2^. At present, eight distinct classes of the soluble cytoplasmatic mammalian GSTs have been identified*: alpha, kappa, mu, omega, pi, sigma, theta,* and *zeta*. The alpha class genes, specifically *GSTA1* are most abundantly expressed in the liver (hepatocytes), kidney (proximal tubules), adrenal glands, pancreas, and testis, while expression in a wide range of other tissues is low^3,4^. Aberrant overexpression has been observed in various malignancies such as colorectal^5^ and lung cancer^6^, while a decrease in alpha class GSTs have been observed in stomach and liver tumours^7^.

In addition to metabolising bilirubin and certain anti-cancer drugs in the liver, GSTs act as modulators of mitogen-activated protein kinase (MAPK) signal transduction pathway via a mechanism involving protein–protein interactions^8–11^. GSTA1 itself has been shown to form complexes with JNK and influences the development of apoptosis^12^.

In terms of pharmacogenomics, GSTA1 holds much importance in the field of oncology, as it is involved in the metabolic pathway of many important chemotherapeutic agents such as busulfan (Bu)^13^, thiotepa^14,15^, doxorubicin^16^, cyclophosphamide^17^, and chlorambucil^18^. Furthermore, because GSTA1 is the most abundantly expressed enzyme of its group in the liver, it establishes itself as the top candidate gene for influencing drug clearance^19^. It is also expressed in the breast, thus thought to be the reason why it influences the efficacy of cyclophosphamide in breast cancer patients^20^. Furthermore, of great interest in onco-haematology is Bu, an alkylating agent used in the conditioning regimen before haematopoietic stem cell transplant (HSCT) as a treatment for different types of malignancies and non-malignancies^8^.

Diverse polymorphisms (SNPs) have been detected after analysis of the proximal promoter of gene encoding *GSTA1*, which is believed to affect its expression. These genetic polymorphisms consist of 2 leading haplotypes, GSTA1*A and GSTA1*B, containing 3 linked base substitutions in the proximal promoter, at positions -52, -69, and -567. Luciferase reporter assays (LRA) showed that GSTA1*A is more highly expressed than GSTA1*B^21^, most likely due to selective binding of Sp1 transcription factor (TF) at positions -52 and -69^21^.

Although most studies in patients receiving Bu based conditioning regimens show positive significant contributions of these haplotypes with Bu pharmacokinetics (PK), adverse events, or disease risk^22–25^, there are still several other studies that have reported negative findings, casting doubt on the role of the promoter SNPs. For a review of *GSTA1*′s role in Bu metabolism see Huezo-Diaz et al.^8^ and for those related to disease-related studies see Deng et al.^26^. The reason for those discrepancies may arise, at least in part, from the incomplete knowledge of *GSTA1* promoter haplotypes distribution in human populations and their impact on *GSTA1* promoter activity, consequently resulting in differences in GSTA1 metabolic potential.

Recently, our group provided additional evidence to suggest that three other SNPs (-513, -631, and -1142) in linkange disequlibrium (LD) with -52, -69, and -567 contribute to the altered GSTA1 promoter activity, enabling the refinement of the haplotypes and thus possibly explaining further the variability between individuals and their impact on Bu metabolism^27^, which could also be true of other substrates of GSTA1.

Thus, with this study, we want to assess the distribution of *GSTA1* haplotypes and diplotypes in different human populations, which can potentially explain different metabolic phenotype distribution among populations. Additionally, this study expands and complements our previous report^27^ by providing further experimental and in silico data aiming to explain the functional contribution of each SNP and to identify TF binding sites encompassing these SNPs.

## Methods

### Population genetics study

We analysed the six SNPs (-52: rs3957356; -69: rs3957357; -513: rs11964968; -567: rs4715332; -631: rs4715333; -1142: rs58912740) genotype data of *GSTA1* promoter from the 1000 Genomes Project Phase 3 Pipeline, Homo sapiens: GRCh37.p13 (GCF_000001405.25) Chr 6 (NC_000006.11), that included 2504 individuals from 26 human populations as described in NCBI Variation Glossary (http://www.ncbi.nlm.nih.gov/variation/docs/glossary). The VCF files containing genotype data from each SNP (rs3957356; rs3957357; rs4715332; rs4715333; rs11964968 and rs58912740) and individual’s ID that identified ethnic group was downloaded and compiled into SPSS. Populations were classified as specified in the 1000 Genome Project into five 5 super-populations that included East Asia, South Asia, Africa, Americas, and Europe. Within each super-population, sub-populations were established that correspond to the country of origin. We used PHASE 2.1.1 software to infer the haplotypes^28^. All SNPs in the article are written in gene-wise 5′ to 3′ orientation. After establishing the haplotypes, we established their frequencies for each super-population and sub-population and compared them using pair-wise FST analysis. Next, diplotype frequencies per super-population were established. Lastly, frequency charts of the metabolic status groups were developed, as previously reported^27^ to evaluate the potential functional impact of *GSTA1* genetic diversity worldwide.

### Distinguishing between A1B1a and A3B2

As PHASE was not able to distinguish the diplotypes *A1 *B1a vs *A3 *B2, we established a reliable PCR-based genotyping method. Forward GSTA1-F-1336BP 5′-*TGGATCCCTCAGTTTTGTAAGG*-3′ and reverse GSTA1-R-1336BP 5′-*TAAACGCTGTCACCGTCC*-3′ oligos were used to specifically amplify the promoter region of *GSTA1* using Platinum SuperFi II PCR Master Mix (ThermoFisher, USA) and the following cycling conditions: initiation for 3 min at 95 °C followed by 38 cycles at 95 °C for 30 s, 64 °C for 30 s and 72 °C for 45 s. PCR products were genotyped using forward and reverse PCR primers and Sanger sequencing service (Fasteris, Switzerland). In the case of ambiguous diplotype, PCR products were TOPO TA cloned using linearized pMiniT 2.0 vector (E1202S, New England Biolabs, USA) according to manufacturer’s recommendations. Several colonies were picked for sequencing using standard SP6 and T7 oligo and *E. coli* NightSeq Sanger sequencing service (Microsynth, Switzerland). This method was validated on a patient showing ambiguous genotype from Ansari et al.^27^ cohort (Table 2) and eight 1000 Genomes Project samples (Supplementary Fig. 2).

### Luciferase reporter gene assay (LRA)

LRA was used to determine how changes to the *GSTA1* promoter sequence due to SNPs affect *GSTA1* promoter function. We constructed 18 different plasmids corresponding to all the haplotypes identified from the population analysis. In brief, the human *GSTA1* promoter (-1430 to -1 nt) was PCR amplified and cloned directly into the SacI-XhoI site of luciferase reporter gene plasmid pGL4.10-basic (Promega, USA). Human hepatoblastoma (HepG2) cells were co-transfected with the pGL4.10 GSTA1 constructs and the pRL-SV40 vector that codes for *Renilla* luciferase for transfection control and normalisation (Promega, USA). Promoterless pGL4.10-basic plasmid was used to determine baseline expression (Promega, USA). Transfections of HepG2 were accomplished by X-tremeGENE HP DNA Transfection Reagent kit (Sigma-Aldrich, USA). Cells were seeded 1 day before transfection and grown for 24 h in 12-well culture plates containing DMEM F12 cell culture medium GlutaMAX supplemented (ThermoFisher, USA). Dual luciferase assay (Promega. USA) and Lumat3 LB 9508 (Berthold Technologies, Germany) were used to measure chemiluminescence as described in the manufacturer’s protocol. Data analysis was performed by normalizing luciferase to renilla chemiluminescence. Next, expression values for each plasmid were compared to the expression of reference *A1 haplotype by calculating the ratio. All measurements were performed at least in triplicates. The experiment was repeated at least three times. The difference in normalized LRA expression was evaluated by paired t-test.

### In silico method for identification of transcription factor binding sites

A non-redundant set of position-weight matrices (PWMs) from binding profiles of known TFs was collected. We used 579 PWMs of vertebrate’s TFs provided from the JASPAR2018 database (v1.1.1, http://jaspar.genereg.net/). Next, using Bedtools (v2.29.2) we extracted the sequence of a 2 kb region around *GSTA1* transcription start site (TSS) from GRCh38.p13 assembly (1.5 kb upstream and 0.5 kb downstream). That region contained the 6 SNPs of interest described above. The sequence was modified to correspond to each haplotype and scanned using HOMER (v4.11). All predictions with a log odd-score higher than 6 were conserved and those having score higher than 10 are presented in Table 3. All haplotype combinations were generated and predictions were sorted by their relative positions to the SNP variation, except for the two nearest SNPs (-52 and -69) from TSS that were packed together due to their proximity.

## Results

### Population genetics study

**Figure 1:**
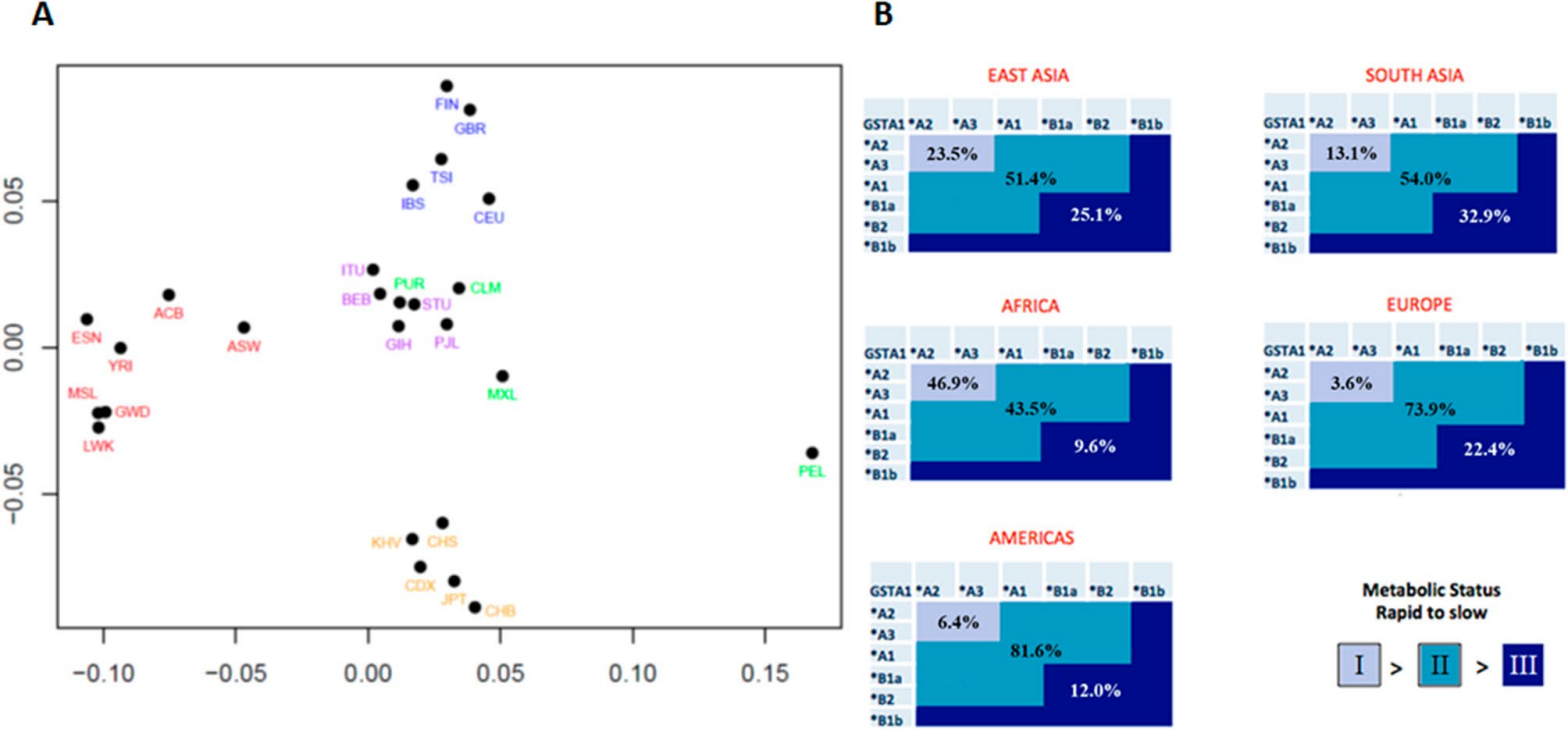
FST analysis of human super- and sub-populations (**A**) and world-wide frequencies of GSTA1 metabolic category (**B**). (**A**) red—African, green—American, light brown—East Asian, blue—European, violet—South Asian sub-populations. (**B**) grey-blue—fast metabolisers, blue—intermediate metabolisers, dark blue—slow metabolisers.

Our data revealed, nine haplotypes (Table 1). Three had not been reported previously, *A1a (GCATTG, N = 4, 0.08%) in Gambian (GWD) population; *B2a (ATATGC, N = 38, 0.76%) in African super-population and *B2b (ATATTG, N = 7, 0.14%) in American, European and South Asian super-populations (Table 1). Haplotype frequencies per sub-population and super-population are illustrated in Table 2. FST analysis (Fig. 1A, Supplementary Fig. 1) shows that all super-populations are homogenous except the American super-population where Peruvians from Lima were significantly different from three other American sub-populations and Mexicans were significantly different from Puerto Ricans. African and East Asian super-population demonstrated significant differences to all other super-populations. European and South Asian super-populations are slightly related but never the less forming clear separate clusters. American super-population is mostly related to South Asian super-population. Finally, using inference, we were not able to determine a diplotype of 8 individuals. Results obtained with our newly established genotyping method demonstrated that genotypes indicated in the 1000 Genomes Project for -52 and -69 were not correct (Supplementary Figs. 2 and 4).

**Table 1:** Nine identified haplotypes in the 1000 Genomes Project population and their composition.

Position	SNP ID	*A1	*A2	*A3	*A1a	*B1a	*B1b	*B2	*B2a	*B2b
-52	rs3957356	G	G	G	G	A	A	A	A	A
-69	rs3957357	C	C	C	C	T	T	T	T	T
-513	rs11964968	A	A	A	A	A	G	A	A	A
-567	rs4715332	T	T	T	T	G	G	G	T	T
-631	rs4715333	T	G	T	G	G	G	G	G	T
-1142	rs58912740	C	C	G	G	G	G	C	C	G

**Table 2:** *GSTA1* Haplotype percentage frequencies for each sub-population and super-population.

Super population	Population code	Population description	*A1	*A2	*A3	*A1a	*B1a	*B1b	*B2	*B2a	*B2b
AFRICAN	ACB	African Caribbeans in Barbados	5.7	58.9	0.0	0.0	5.2	0.0	27.6	2.6	0.0
	ASW	Americans of African Ancestry in SW USA	9.8	57.4	0.0	0.0	9.8	1.6	19.7	1.6	0.0
	ESN	Esan in Nigeria	0.0	66.2	0.0	0.0	2.5	0.0	27.3	4.0	0.0
	GWD	Gambian in Western Divisions in the Gambia	0.0	69.0	0.0	2.7	3.1	0.0	23.5	1.8	0.0
	LWK	Luhya in Webuye, Kenya	1.5	69.2	0.0	0.0	1.0	0.0	25.3	2.5	0.5
	MSL	Mende in Sierra Leone	0.0	70.8	0.0	0.0	3.0	0.0	22.0	4.2	0.0
	YRI	Yoruba in Ibadan, Nigeria	0.5	68.1	0.0	0.0	6.5	0.0	22.2	2.8	0.0
	Average		0.5	68.2	0.0	0.0	6.5	0.0	22.0	2.8	0.0
AMERICAN	CLM	Colombians from Medellin, Colombia	42.0	21.8	0.0	0.0	26.6	5.9	2.7	0.0	1.1
	MXL	Mexican Ancestry from Los Angeles USA	47.7	27.3	0.0	0.0	25.0	0.0	0.0	0.0	0.0
	PEL	Peruvians from Lima, Peru	68.2	12.4	0.0	0.0	17.1	0.6	1.2	0.0	0.6
	PUR	Puerto Ricans from Puerto Rico	35.6	28.8	0.0	0.0	28.4	1.4	5.3	0.5	0.0
	Average		47.5	22.7	0.0	0.0	24.4	2.2	2.6	0.1	0.4
EAST ASIAN	CDX	Chinese Dai in Xishuangbanna, China	33.9	52.7	0.0	0.0	0.0	13.4	0.0	0.0	0.0
	CHB	Han Chinese in Bejing, China	39.8	48.1	0.0	0.0	1.5	10.7	0.0	0.0	0.0
	CHS	Southern Han Chinese	37.6	47.1	0.0	0.0	0.5	14.8	0.0	0.0	0.0
	JPT	Japanese in Tokyo, Japan	38.0	49.0	0.0	0.0	0.0	13.0	0.0	0.0	0.0
	KHV	Kinh in Ho Chi Minh City, Vietnam	33.3	52.0	0.0	0.0	0.5	14.1	0.0	0.0	0.0
	Average		36.6	49.9	0.0	0.0	0.5	13.0	0.0	0.0	0.0
EUROPEAN	CEU	Utah Residents (CEPH)	43.9	15.7	0.0	0.0	35.4	5.1	0.0	0.0	0.0
	FIN	Finnish in Finland	34.3	19.7	0.0	0.0	33.3	11.6	0.5	0.0	0.5
	GBR	British in England and Scotland	38.5	17.0	0.0	0.0	37.9	5.5	0.5	0.0	0.5
	IBS	Iberian Population in Spain	33.0	25.5	0.0	0.0	36.8	3.3	0.9	0.0	0.5
	TSI	Toscani in Italia	36.9	21.0	0.0	0.0	40.7	1.4	0.0	0.0	0.0
	Average		37.3	19.9	0.0	0.0	36.9	5.3	0.4	0.0	0.3
SOUTH ASIAN	BEB	Bengali from Bangladesh	26.7	38.4	0.0	0.0	16.9	18.0	0.0	0.0	0.0
	GIH	Gujarati Indian from Houston, Texas	32.0	35.0	0.5	0.0	24.3	8.3	0.0	0.0	0.0
	ITU	Indian Telugu from the UK	25.5	37.7	0.0	0.0	17.6	18.6	0.5	0.0	0.0
	PJL	Punjabi from Lahore, Pakistan	40.0	27.4	0.0	0.0	17.4	15.3	0.0	0.0	0.0
	STU	Sri Lankan Tamil from the UK	33.2	32.2	0.0	0.0	14.9	19.8	0.0	0.0	0.0
	Average		31.5	34.1	0.1	0.0	18.3	15.9	0.1	0.0	0.0

The frequencies are based on the following numbers: Africa, N = 660; America, N = 347; East Asia, N = 504; European, N = 502; South Asia, N = 488.In populations of Europe, South Asia, and America the frequency of *A3 and *B2 is probably underestimated on account of *A1*B1a.

**Table 3:** *GSTA1* diplotype frequency data per super population.

Metabolic group	Diplotype	Ansari et al. % (N)		Africa % (N)		Americas % (N)		East Asia % (N)		Europe % (N)		South Asia % (N)	
1	A2A2	8.8	(12)	43.5	(287)	6.3	(22)	23.4	(118)	3.6	(18)	13.1	(64)
	A2A3	0.7	(1)	0.0	(0)	0.0	(0)	0.0	(0)	0.0	(0)	0.0	(0)
2	A1A1	12.4	(17)	0.2	(1)	22.8	(79)	12.3	(62)	13.5	(68)	11.9	(58)
	A1A2	13.1	(18)	2.6	(17)	21.0	(73)	38.3	(193)	14.7	(74)	19.1	(93)
	A1B1a or *A3*B2	35.0	(48)	0.0	(0)	24.2	(84)	0.0	(0)	28.9	(145)	9.8	(48)
	A1B2	0.7	(1)	1.2	(8)	2.0	(7)	0.0	(0)	0.0	(0)	0.0	(0)
	A2A1a	0.0	(0)	0.8	(5)	0.0	(0)	0.0	(0)	0.0	(0)	0.0	(0)
	A2B1a	8.0	(11)	5.5	(36)	9.8	(34)	0.6	(3)	16.3	(82)	13.1	(64)
	A2B2	7.3	(10)	31.7	(209)	0.9	(3)	0.0	(0)	0.0	(0)	0.0	(0)
	A2B2a	0.0	(0)	4.5	(30)	0.3	(1)	0.0	(0)	0.0	(0)	0.0	(0)
	A2B2b	0.0	(0)	0.2	(1)	0.0	(0)	0.0	(0)	0.0	(0)	0.0	(0)
	A3B1a	0.0	(0)	0.0	(0)	0.0	(0)	0.0	(0)	0.0	(0)	0.2	(1)
3	A1B1b	1.5	(2)	0.0	(0)	2.3	(8)	10.3	(52)	3.8	(19)	10.5	(51)
	A2B1b	2.2	(3)	0.2	(1)	0.6	(2)	13.7	(69)	1.6	(8)	9.6	(47)
	A1aB1b	0.0	(0)	0.2	(1)	0.0	(0)	0.0	(0)	0.0	(0)	0.0	(0)
	B1aB1a	7.3	(10)	0.3	(2)	6.3	(22)	0.0	(0)	12.0	(60)	3.9	(19)
	B1aB1b	2.2	(3)	0.0	(0)	1.2	(4)	0.4	(2)	4.4	(22)	5.5	(27)
	B1aB2	0.7	(1)	2.1	(14)	1.2	(4)	0.0	(0)	0.2	(1)	0.2	(1)
	B1bB1b	0.0	(0)	0.0	(0)	0.0	(0)	1.0	(5)	0.4	(2)	3.1	(15)
	B1bB2	0.0	(0)	0.2	(1)	0.3	(1)	0.0	(0)	0.0	(0)	0.0	(0)
	B2B2	0.0	(0)	6.1	(40)	0.0	(0)	0.0	(0)	0.0	(0)	0.0	(0)
	B2B2a	0.0	(0)	1.1	(7)	0.0	(0)	0.0	(0)	0.0	(0)	0.0	(0)
	B2B2b	0.0	(0)	0.0	(0)	0.9	(3)	0.0	(0)	0.6	(3)	0.0	(0)

**Figure 2:**
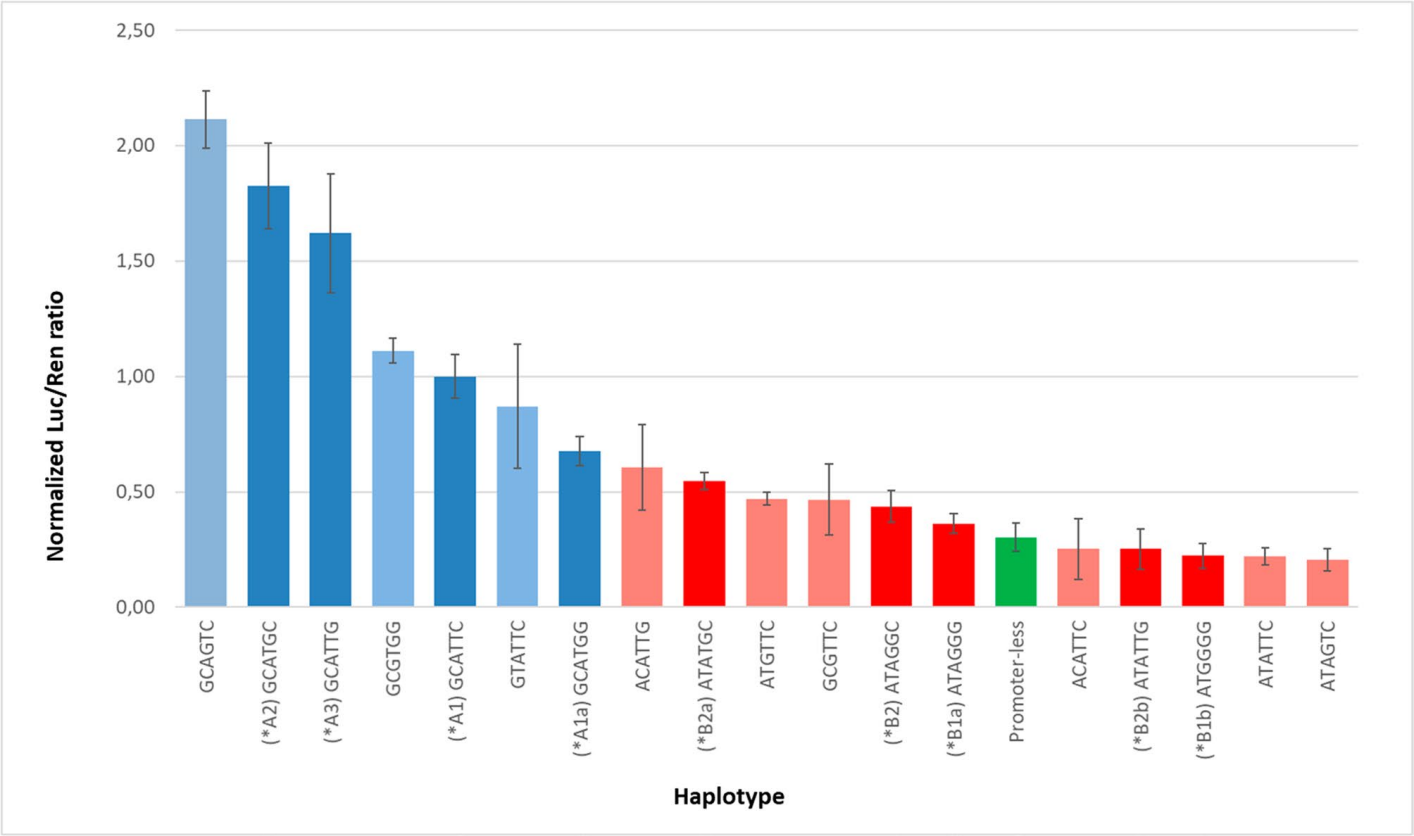
Luciferase reporter assay results for each haplotype. Dark blue (*A haplotypes), dark red (*B haplotypes), faded colours (non-existent haplotypes), green (no promoter).

In terms of diplotypes from the 45 possible combinations, we detected 24, including 6 combinations with newly reported haplotypes: *A2*A1a (N = 4, 0.16%); *A2*B2a (N = 31, 1.24%); *A2*B2b (N = 1, 0.04%); *A1a*B1a (N = 1, 0.04%), *B2*B2a (N = 7, 0.28%) and *B2 *B2b (N = 6, 0.24%). Most of these diplotypes were identified in individuals of African ancestry (Table 3).

In our previous study^29^, we have determined three functional *GSTA1* metabolic phenotypes based on the combinations of the identified six haplotypes. Using the same categorization, the distribution of these metabolic phenotypes demonstrates clear ethnic differences (Fig. 1B). The frequency of the fast metaboliser phenotype (group 1) is highest in the African super-population (46.9%), followed by East Asian (23.5%), South Asian (13.1%), American (6.4%), and European super-population (3.6%). On the other hand, the frequency of the slow metaboliser phenotype is highest in the South Asian super-population (32.9%) followed by East Asian (25.1%), European (22.4%), American (12.0%), and African (9.6%) super-populations.

The reason for the high frequency of slow metaboliser phenotype in East and South Asians is due mostly to the higher frequency of *B1b haplotype (13.0% and 15.9%, respectively), whereas in the European super-population slow metabolisers carry mostly *B1a and *B1b haplotypes (allele frequency of 36.9% and 5.3%, respectively) (Tables 2 and 3).

### Reporter gene assay

**Figure 3:**
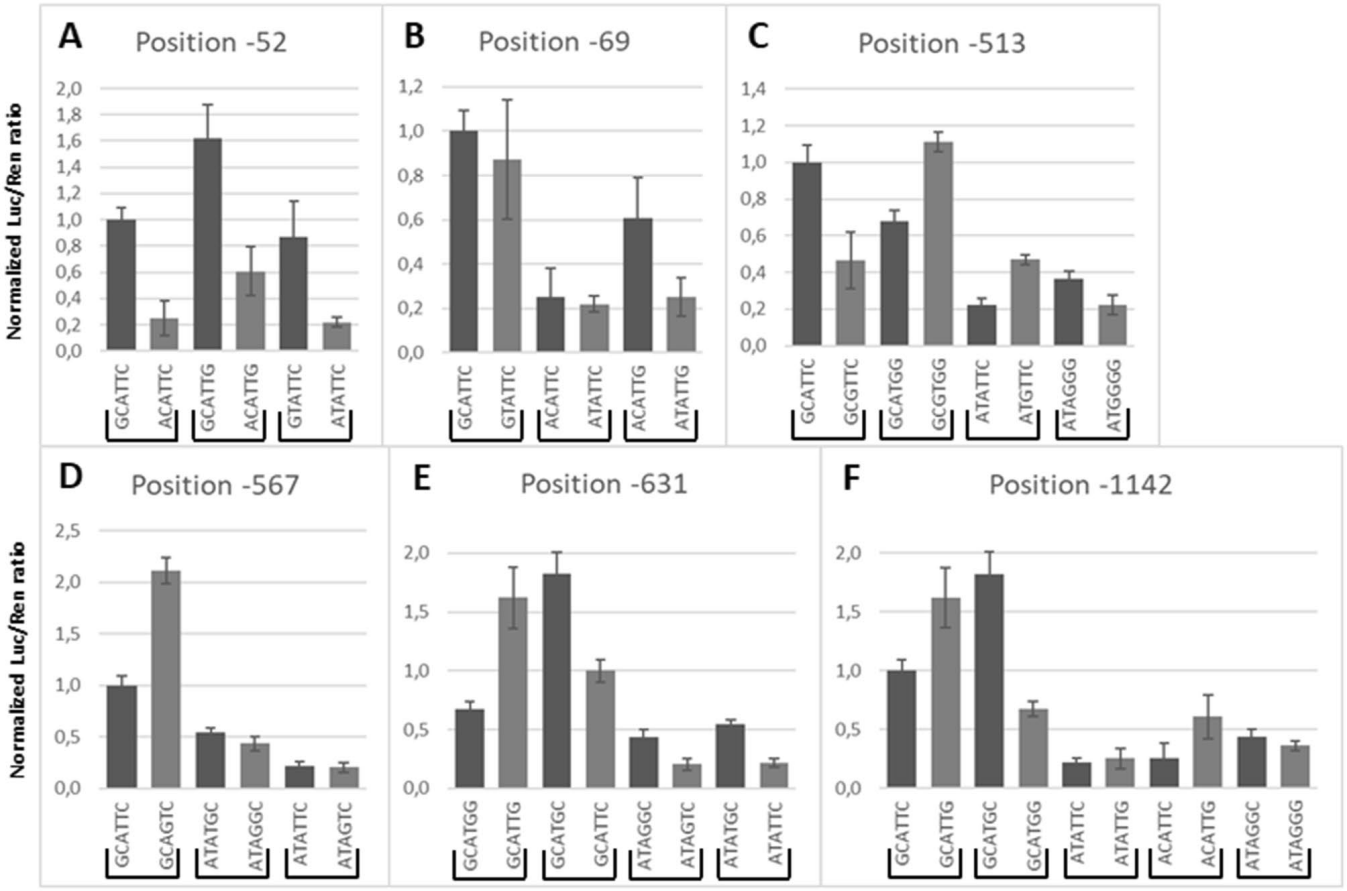
Luciferase reporter assay results grouped by position. The impact of SNPs was evaluated by pairwise comparison for each SNP site. Results of plasmid LRA were paired per site of change and identity of surrounding haplotype. The results demonstrate that surrounding haplotypes can have an impact on the functioning of the SNP, therefore, indicating an interaction between two sites (see sections "Results", "Reporter gene assay" and "Discussion").

To test the effects of each SNP on promoter activity, we performed LRAs using different haplotype constructs (Figs. 2 and 3). The results confirm previous observations that *A haplotypes express luciferase consistently better than *B haplotypes (Fig. 2). All of the new haplotypes identified in this study, *A1a, *B2a, and *B2b, demonstrated significant expression differences to their counterparts, as shown in Fig. 2; *A1a vs *A2 (p = 0.055) or *A3 (p = 0.007); *B2a vs *B2 (p = 0.002) and *B2b vs *B2 (p = 0.02). Next, we investigated how these six SNPs interact with each other to modulate *GSTA1* expression (Fig. 3A–F).

#### -52 and -69 (rs3957356 and rs3957357)

Present results suggest that position -52 is more important for the functioning of the leading haplotype in comparison to -69. Also, the position -1142 interacts with both -52 and -69 SNPs (Fig. 3A and B). Comparing plasmids GCATTC (*A1), GCATTG, and GTATTC against ACATTC, ACATTG, and ATATTC, respectively, confirmed that G > A SNP at position -52 determines the main functional effect of the *GSTA1* haplotypes. Luciferase expression decreased by 3.96-fold (p < 0.001), 2.67-fold (p < 0.001) and 3.95-fold (p = 0.016), respectively, when variant A was present. This result suggests that the SNP at position -1142 could have an impact on -52 because in the context of G at -1142 the decrease in luciferase expression was lower than in the presence of C at the same -1142 site. To verify the leading role of SNP at -52, we investigated the effect of C > T SNP at position -69. Luciferase expression decreased only by 1.15-fold (p = 0.003) when changing from GCATTC (*A1) to GTATTC. The same 1.15-fold (p = 0.06) difference in expression was observed in the context of *B haplotype (ACATTC vs ATATTC), although the result was not statistically significant. Interestingly, SNP at position -1142 demonstrated a significant effect on SNP at position -69 as seen from the 2.40-fold (p < 0.001) decrease when comparing ACATTG to ATATTG.

#### -513 (rs11964968)

SNP located at -513 is in LD with -52 and -69 and is known to appear only in *B haplotypes (Table 2). Nevertheless, we investigated its behaviour using LRA in the context of haplotype *A to better understand its function. The results suggest that SNP at -513 interacts with -52/-69, -631, and -1142 SNPs to modulate the expression of the promoter (Fig. 3C). The A > G SNP at position -513 caused a 1.63-fold (p = 0.08) decrease in the context of *B haplotype, visible when haplotypes ATAGGG (*B1a) and ATGGGG (*B1b) were compared. Interestingly however, ATGTTC demonstrated 2.13-fold (p = 0.001) higher expression versus ATATTC. In the context of the *A haplotype, the pattern was reversed. The change A > G resulted in 2.15-fold (p = 0.005) lower expression of GCGTTC versus GCATTC (*A1) but in 1.64-fold (p = 0.005) higher expression in GCGTGG versus GCATGG (*A1a).

#### -567 (rs4715332)

The fourth SNP, T > G at position -567 is also in LD with -52 and -69 and is known to appear only in *B haplotypes. Results suggest that -567 also interacts with -52/-69 (Fig. 3D). In the context of *B haplotypes, which were detected as a small minority in the 1000 Genomes Project population, ATATGC (*B2a) resulted in a 1.25-fold (p = 0.04) higher expression versus ATAGGC (*B2). A similar low impact from this SNP was observed in an ATATTC vs ATAGTC pair (1.05-fold, p = NS). In the context of *A haplotype, a T to G change resulted in a dramatic increase of luciferase expression (GCATTC vs GCAGTC, 2.11-fold, p = 0.02) superseding even *A2 expression levels suggesting that position -567 interacts with the leading (-52, -69) positions but not with position -631.

#### -631 (rs4715333)

The results suggest that the -631 site is independent of the -52/-69 but dependent on the changes at -1142 (Fig. 3E). As previously shown, changing T > G at SNP location -631 GCATTC (*A1) vs GCATGC (*A2) resulted in 1.82-fold (p = 0.01) higher luciferase expression in the context of haplotype *A. The same result is observed in the context of the *B haplotype. ATATGC (*B2a) has a 2.5-fold (p = 0.04) higher expression in comparison to ATATTC and the same is true when comparing ATAGGC (*B2) to ATAGTC (2.09-fold, p = 0.007)**.** Interestingly, in the context of the SNP at -1142, we observed an inverse relationship. GCATGG has a 2.40-fold (p = 0.007) lower expression than GCATTG while GCATGC has a 1.82-fold (p = 0.046) higher expression than GCATTC.

#### -1142 (rs58912740)

The results for -1142 further support the above-described interaction between the -52/-69 and the -1142 SNP (Fig. 3F). The C to G change at position -1142 resulted in a 1.62-fold (p = 0.002) higher expression when analysed in the context of haplotype *A (GCATTC (*A1) vs GCATTG (*A3)) while an inverse relationship was found when comparing GCATGC (*A2) to GCATGG (*A1a), whereby a 2.70-fold (p = 0.014) decrease in expression resulted when—631 was also changed. In the context of the *B haplotype, we observed the repetition of the expression pattern when -631 was taken into consideration. However, the differences in expression were much smaller suggesting an interaction between the -52/-69 and the -1142 site. We observed a 1.20-fold (p > 0.5) increase of expression between ATAGGG (*B1a) and ATAGGC (*B2), while a 1.14-fold (p = 0.06) increase between ATATTC and ATATTG. Interestingly, ACATTC demonstrated a 2.40-fold (p = 0.001) increase compared to ACATTG.

**Figure 4:**
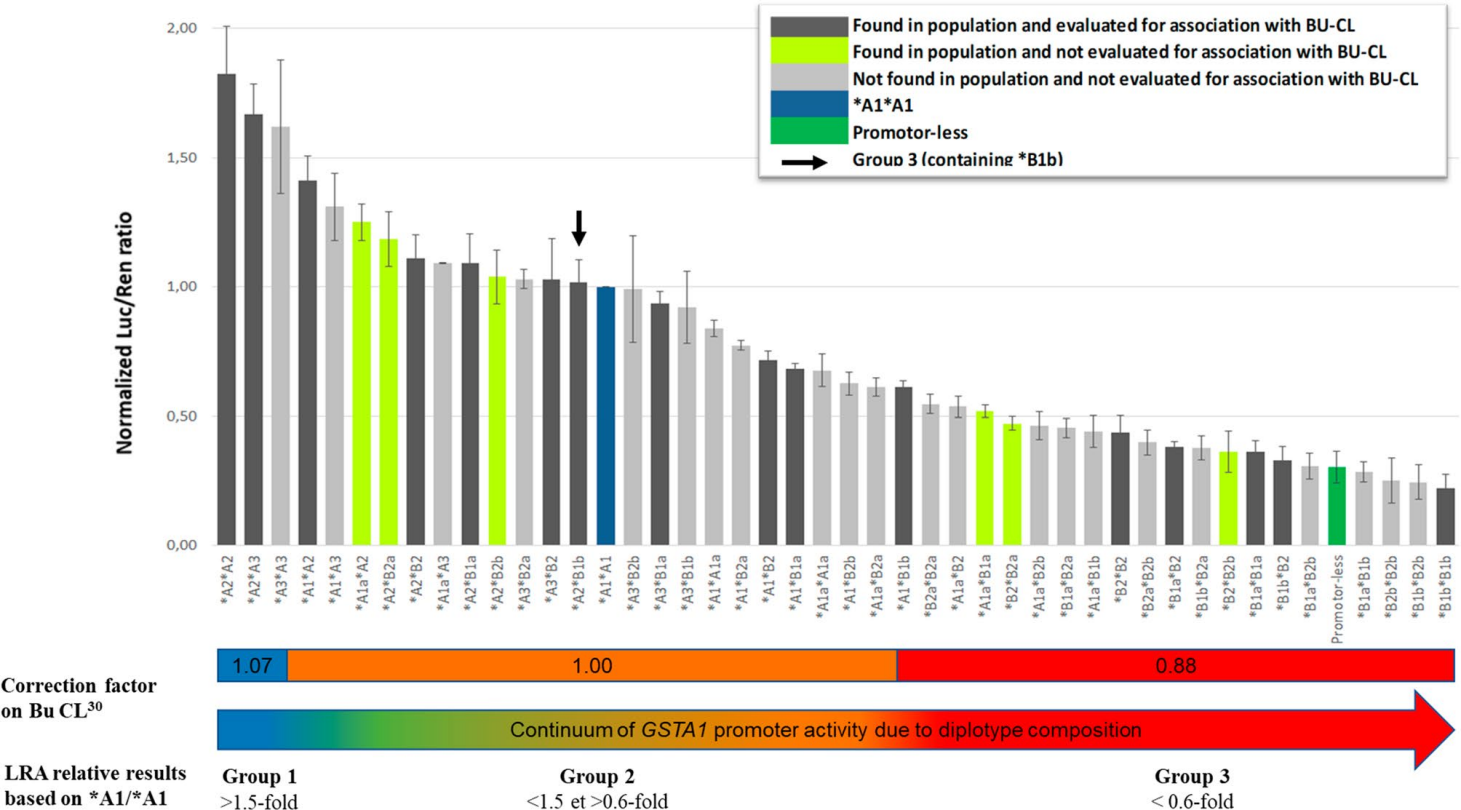
Predicted GSTA1 diplotype expression levels.

Expected diplotype functions, shown in Fig. 4, are predicted by calculating the mean expression activity. The additive model was based on the measurement of luciferase expression of equimolar mixtures. Results confirmed the additive relationship between haplotypes (Supplementary Fig. 3). Out of 45 possible diplotype combinations of nine haplotypes, we detected 23 diplotypes in the 1000 Genomes Project population. Seventeen were reported previously in a Bu pharmacokinetic association study^27^, which were subsequently incorporated into a population pharmacokinetic model whereby two extreme groups of diplotypes carried by around 30% of the analysed population were confirmed of having extreme metabolic potential in comparison with the reference^30^ (Fig. 4).

Considering the classification in those three Bu-based metabolic groups, calculated LRA expressions showed that group 1 (fast metabolisers) present around 1.5-fold higher expression than the reference diplotype *A1*A1, whereas group 3 (slow metabolisers) have around 1.6-fold lower expression than the reference (Fig. 4). Based on our results, new diplotypes (in green) *A1a*A2, *A2*B2a and *A2*B2b are likely to be placed in the intermediate metabolic group while *B1a*B2b, *B2*B2a, and *B2*B2b are most likely resulting in slow GSTA1 activity. Based on the expected LRA expressions, diplotypes containing *B1b haplotype (*A2*B1b and *A3*B1b) are the only diplotypes that did not follow the GSTA1 metabolic categorization, as previously described^27^ (Fig. 4).

Twenty-two diplotypes were not detected in the 1000 Genomes Project population (light grey) due to the low haplotype frequencies and were also not evaluated for association with BU-clearance. Using LRA results we predicted their ability to express *GSTA1*. Only *A3*A3 would be placed in the high Bu-metabolising group. Seven diplotypes (*A1*A3, *A1a*A3, *A3*B2a, *A3*B2b, *A3*B1b, *A1*A1a, *A1*B2a) would be placed in intermediate BU-metabolising group (Group 2). Three diplotypes (between *A1*B1a and *A1*B1b): *A1a*A1a, *A1*B2b, and *A1a*B2a are borderline between groups 2 and 3, while the remaining eleven diplotypes are in the slow Bu-metabolising group 3. Group 3 is composed exclusively of *B haplotypes or the lowest expressing form of *A haplotype *A1a. The only exception is *A1*B1b containing *B1b previously associated with slow metaboliser status^27^.

### In silico method for identification of transcription factor binding sites (DNA–protein)

Using in silico analysis, promoter region encompassing all SNPs’ positions were analysed for binding of putative TFs (Table 4 and Supplementary Fig. 5). Because -52 and -69 sites are close together, we chose to analyse both locations together as either GC (*A) or TA (*B). The analysis identified RARA, E2F6, Sp1, NR2F1, and KLF5 as potentially binding to this location in the case of GC haplotype. Particularly, the detection of Sp1 was important as Sp1 has previously demonstrated to bind to this position. In the case of haplotype TA, only the NR2E1 binding motif was found to have a high similarity to this position. Position -513 was the most variable position out of all six positions. Fourteen different TFs from the FOX family, 4 TFs from Sox, 2 TFs from HOX and CDX2 families were predicted to bind to this site in the case of the A allele, whereas no unique TFs were found to bind when the G allele was present (*B1b). Four TFs were found to bind to both alleles but again preferentially to the A allele with a score difference of more than 4: 2 HOX family TFs, FOXF2, and CDX1. At position -567, it was found that only GATA1 exhibited high binding capacity. The score difference of -5.451 suggests that the G allele is more similar to the consensus GATA binding sequence in comparison to the T allele. Predictions at position -631 suggested binding of PRDM1 and IRF1 but allele-specific binding scores were almost identical. All other TFs had a score lower than 10. It is of note nevertheless that there are possibly 13 TFs binding to the G allele in comparison only to 2 in the T allele. At position -1142 only 2 potential TFs were predicted to bind to the G allele (NR1A4 and NFIC) but had a low score and no TFs were found for the C allele.

**Table 4:** Prediction of TFs binding to 6 different SNP sites. TFs with a score of more than 10 or a difference in score of more than 4 are shown.

Position	pwmname	Allel	Score	Allel	Score
-52 / -69	RARA::RXRG	GC	13.40	AT	
	E2F6	GC	12.22	AT	
	SP1	GC	11.21	AT	
	NR2F1	GC	10.96	AT	
	Pparg::Rxra	GC	10.09	AT	
	KLF5	GC	10.06	AT	
	Nr2e1	GC		AT	11.79
-513	CDX1	G	6.08	A	10.80
	HOXA13	G	6.21	A	10.96
	FOXF2	G	7.10	A	14.18
	Hoxd8	G	7.68	A	12.18
	Foxj3	G		A	14.19
	Foxj2	G		A	13.58
	FOXG1	G		A	13.54
	FOXL1	G		A	12.01
	FOXP2	G		A	11.98
	Sox5	G		A	11.92
	SOX15	G		A	11.89
	SRY	G		A	11.79
	Foxq1	G		A	11.67
	CDX2	G		A	11.45
	FOXK2	G		A	11.21
	Hoxc9	G		A	11.20
	FOXO4	G		A	11.12
	FOXO6	G		A	11.06
	Hoxa9	G		A	10.77
	FOXC2	G		A	10.77
	Foxq1	G		A	10.69
	FOXK1	G		A	10.29
	FOXI1	G		A	10.25
	FOXO3	G		A	10.21
	SOX9	G		A	10.18
-567	GATA1::TAL1	T	10.76	G	16.21
-631	PRDM1	G	11.25	T	10.18
	IRF1	G	10.79	T	11.51

## Discussion

In the present study, we have analysed the distribution of the *GSTA1* promoter haplotypes in varying human populations to understand how ethnicity could impact the GSTA1 functional profile. Six SNPs that were previously investigated for functional impact on the GSTA1 function were selected for this study^4,27,30–32^. We show that there are significant differences between human populations with regards to their potential functionality to express *GSTA1* as assessed by LRA.

Although the population data demonstrate a very similar distribution of the GSTA1*A and GSTA1*B across the populations, a significant difference was apparent when a more detailed analysis of the additional SNPs, located in the *GSTA1* promoter region, were included in the haplotype analyses. The results suggest that the genotyping of all six polymorphisms in the *GSTA1* promoter should be performed in some populations but may not be crucial for others.

One such example where genotyping only for -52/-69 position could be sufficient is African super-population which is fairly homogenous, composed mainly of *A2 (representing 96% of all *A haplotypes) and *B2 (representing 76% of all *B haplotypes) which altogether represent 89,6% of all haplotypes. Nevertheless, even in such a population, a significant number of individuals could be mischaracterized due to the presence of other less frequent alleles. In contrast, in American, European, and South Asian super-populations, the variations of *A and *B are more frequent, leading to diverse diplotype possibilities of potentially very different GSTA1 functionality, making the functional prediction of GSTA1 through promoter genotyping more challenging. East Asian super-population is particular because it has a homogenous frequency of *B1b which accounts for more than 96% of all *B haplotypes. Thus, the detection of one haplotype *B, regardless of the diplotype, will result in poor Bu metabolisation since *B1b has been associated with low expression even in combination with *A^27^. Nevertheless, *A represents 86.5% of all haplotypes and is very diverse in this population, thus the genotyping of positions -631 and -1142 is still necessary to distinguish between rapid and intermediate metabolisers in *A homozygous individuals.

These findings could explain some discordant results reported by other groups concerning the association of Bu clearance and *GSTA1* *A/*B haplotypes through population PK modeling. Zwaveling et al.^33^, in a study on children mostly of European origin, failed to detect any association between *GSTA1* promoter haplotypes and the clearance of Bu based only on the *A/*B haplotypes^33^. In contrast, Choi et al.^34^, reported a 15% decrease of Bu clearance in Korean adults carrying at least one *B haplotype^34^. Results reported in this study, suggest that the Korean patients genotyped as *B may have a *B1b haplotype. This haplotype is consistently associated with the lowest expression of *GSTA1*, according to our LRA results. On the other hand, in the European super-population, the most frequent *B haplotype is *B1a (88% of all *B) which has a 1.63-fold higher expression than *B1b. In contrast to *B1b, *B1a leads to a poor *GSTA1* expression only when in combination with another *B haplotype, otherwise the combinations with the most common *A haplotypes of European super-population result in an intermediate Bu metabolising capacity.

Although the majority of diplotypes can be deduced with high probability after the use of common genotyping methods, there is a possibility of diplotype mischaracterization given a large number of theoretically possible diplotypes. The most notable example is ambiguity between *A1*B1a and *A3*B2. Even though both diplotypes fall into the same metabolic group with regards to Bu clearance, they were significantly different when compared with LRA and might result in being part of different metabolic groups when other drugs are involved. Our newly optimized genotyping protocol demonstrated that in case of ambiguous or potentially faulty genotyping results real diplotypes could be obtained with ease and without the need to rely on inference (Supplementary Fig. 3).

Using different variations of the *GSTA1* promoter regions, we were able to infer the functional impact of these differences and establish a new model of TF interactions based on these results. As demonstrated in our previous research the GSTA1 metabolic profile as assessed by LRA will not necessarily be completely concordant with metabolic groups established through the use of in vivo data (Figs. 1B and 4 – *B1b combinations with *A haplotypes). Nevertheless, as demonstrated in our previous publications^27,29,30^, three Bu-based metabolic groups that tightly follow LRA stratification of *GSTA1* could be established using PK data of Bu clearance in humans.

Lastly, the data from LRA allowed us to better understand the functioning of the *GSTA1* promoter region. As demonstrated in the LRA results and as published previously the *A/*B haplotype determines the strength of the promoter^21^. However, other sites (-513, -567, and -1142) interact with the -52/-69 position and in some cases between each other. Of particular interest is the SNP at site -1142, which appears to be modulated by both -631 and by the -52/-69 and site -513 which is modulated by -1142 and by the -52/-69. On the other hand, the -567 position seemed to be of lesser importance. Previous research demonstrated that -52/-69 is a site of Sp1 TF^21^. Sp1 is known to be able to partner with other TFs^35^. To understand which other TFs could be bound to these sites, in silico predictions were performed. Consistent with previous research, we identified an Sp1 binding site at position -52/-69. In accordance with LRA results, GC (*A) allele appears to bind more TFs (6) than the AT allele (*B) (1). Some of those like E2F6 suggest the presence of a nearby transcription start site and may not be present if there is an AT allele pointing to the reason why AT allele (*B haplotypes) in general expresses *GSTA*1 at a lower rate^36^. In our model, site -513 is predicted to interact with the -52/-69 and with -1142. Curiously, the in silico prediction demonstrates a wide range of possible TFs binding at this location. The most notable appears to be TFs from FOX, Hox, Sox, and CDK family. The DNA sequence of an A allele appears to be more similar to the consensus sequences of all 4 families. The least explored site -567 which is in strong LD with the -52/-69, presents itself as a possible GATA binding position. The results of the LRA suggest that the -567 site is in strong co-operation with the -52/-69 possibly through the interaction of Sp1 and GATA1^35^. This interaction could explain why there is a further boost to transcription activity in the context of *A haplotype (GCATTC vs GCAGTC) but not *B haplotypes (Fig. 3D). In addition to GATA1, G and T alleles are both predicted to bind 6 unique TFs, however, due to the lower score, it is questionable if this finding could have any functional significance. The last two positions, -631 and -1142, that interact with each other demonstrated no clear TF predictions for -1142 apart from NR1A4 and NFIC that demonstrated only low similarity in presence of G allele. Interestingly, the G allele at site -631 had a higher number of predictions in comparison to T allele, which demonstrated similarity to only 2 unique TFs. In addition to the interaction between each other TFs at the -1142 site appears to be in strong co-operation with the -52/-69 and above discussed -513 site. On the other hand, changing the -52/-69 haplotype or -567 does not appear to change the functioning of -631 suggesting that -631 does not co-operate with those two sites.

It has to be noted that there are other SNPs present in the *GSTA1* promoter region. Particularly, the African super-population is genetically diverse. It has at least 4 SNPs which have a frequency of more than 4.5% that are very rare or absent in all other human populations (rs3996998, rs9296692, rs3756985, rs56320607). Our approach was to include globally more frequent SNPs in our analysis for which functionality had been previously assessed. Although scientifically relevant, the inclusion of population-specific SNPs would lead to the prohibitively high number of reporter plasmid combinations. In addition to the above-mentioned limitations, it remains unclear why individuals with *B1b haplotype demonstrated low Bu-clearance. One explanation could be that the additive model of haplotypes does not reflect the reality in the case of *B1b and that there is an interaction between 2 alleles.

In conclusion, this study demonstrates a clear ethnic difference in the promoter region of this important pharmaco-gene: *GSTA1*. We were able to identify new *GSTA1* promoter haplotypes, characterize their impact on expression by using LRA, and predict their metabolic impact. A new model of interaction of six polymorphic positions was established with a clear interaction between positions -52/-69, -513, and -1142. While the -52/-69 Sp1 binding element remains the most important determinant of *GSTA1* expression, it has to be complemented with the genotyping of additional SNPs to determine a metabolic status even if the ethnicity of the individual is known. We established a specific and optimized method for reliable haplotyping of *GSTA1* promoter that does not rely on computational inference but rather precise molecular characterization through cloning. Lastly, the data in the current paper represent the opportunity to establish similar metabolic groups for all drugs metabolised by GSTA1.

## Supplementary Information

Supplementary information.
