# Supplementary material for: The analysis of GSTA1 promoter genetic and functional diversity of human populations
Source: Sci Rep. 2021 Mar 3;11:5038. doi: 10.1038/s41598-021-83996-2 (PMC7930039; doi:10.1038/s41598-021-83996-2)
Supplement: Supplementary file 1 — Supplementary information. [file 41598_2021_83996_MOESM1_ESM.pdf]

The analysis of GSTA1 promoter genetic and functional diversity of human populations

Vid Mlakar, Patricia Huezo-Diaz Curtis, Marc Armengol, Victor Ythier, Isabelle Dupanloup, Khalil Ben Hassine, Laurence Lesne, Rabih Murr, Simona Jurkovic Mlakar, Tiago Nava, Marc Ansari

Supplementary Material

Supplementary Figure 1. Pairwise FST analysis of human sub-populations - distances

			African							European					East Asian					South Asian					American			
			ACB	ASW	ESN	GWD	LWK	MSL	YRI	CEU	FIN	GBR	IBS	TSI	CDX	CHB	CHS	JPT	KHV	BEB	GIH	ITU	PJL	STU	CLM	MXL	PEL	PUR
			1	2	3	4	5	6	7	8	9	10	11	12	13	14	15	16	17	18	19	20	21	22	23	24	25	26
African	ACB	1	0,000																									
	ASW	2	-0,002	0,000																								
	ESN	3	-0,001	0,006	0,000																							
	GWD	4	0,007	0,007	0,001	0,000																						
	LWK	5	0,006	0,009	0,000	-0,003	0,000																					
	MSL	6	0,007	0,009	0,000	-0,004	-0,005	0,000																				
	YRI	7	0,000	0,002	-0,003	-0,003	-0,001	-0,003	0,000																			
European	CEU	8	0,117	0,085	0,152	0,159	0,164	0,161	0,144	0,000																		
	FIN	9	0,119	0,090	0,153	0,164	0,171	0,166	0,146	0,004	0,000																	
	GBR	10	0,121	0,090	0,156	0,167	0,173	0,169	0,149	-0,002	-0,003	0,000																
	IBS	11	0,092	0,065	0,124	0,131	0,138	0,135	0,116	0,000	0,001	-0,002	0,000															
	TSI	12	0,106	0,077	0,139	0,147	0,153	0,150	0,131	-0,002	0,001	-0,003	-0,004	0,000														
East Asian	CDX	13	0,126	0,091	0,142	0,117	0,120	0,120	0,125	0,125	0,158	0,153	0,129	0,139	0,000													
	CHB	14	0,151	0,115	0,170	0,145	0,147	0,148	0,153	0,135	0,175	0,167	0,144	0,152	-0,002	0,000												
	CHS	15	0,124	0,089	0,143	0,122	0,124	0,124	0,127	0,107	0,142	0,135	0,115	0,123	-0,003	-0,001	0,000											
	JPT	16	0,140	0,104	0,158	0,134	0,136	0,137	0,141	0,127	0,165	0,158	0,135	0,144	-0,004	-0,004	-0,003	0,000										
	KHV	17	0,117	0,082	0,133	0,109	0,112	0,112	0,116	0,115	0,147	0,142	0,119	0,128	-0,005	0,000	-0,004	-0,003	0,000									
South Asian	BEB	18	0,071	0,042	0,096	0,096	0,104	0,100	0,086	0,017	0,016	0,020	0,012	0,018	0,080	0,100	0,069	0,089	0,071	0,000								
	GIH	19	0,069	0,040	0,095	0,093	0,099	0,097	0,084	0,011	0,022	0,018	0,008	0,013	0,065	0,078	0,054	0,070	0,057	0,001	0,000							
	ITU	20	0,072	0,045	0,096	0,099	0,106	0,103	0,088	0,018	0,013	0,017	0,010	0,017	0,092	0,112	0,081	0,101	0,082	-0,005	0,004	0,000						
	PJL	21	0,092	0,060	0,121	0,121	0,125	0,123	0,111	0,009	0,022	0,019	0,013	0,017	0,065	0,075	0,051	0,068	0,057	0,003	-0,001	0,006	0,000					
	STU	22	0,084	0,054	0,110	0,111	0,116	0,113	0,101	0,013	0,016	0,018	0,013	0,018	0,075	0,091	0,063	0,081	0,066	-0,004	0,001	-0,002	-0,002	0,000				
American	CLM	23	0,096	0,065	0,129	0,132	0,136	0,134	0,120	-0,001	0,016	0,008	0,005	0,005	0,087	0,096	0,072	0,089	0,079	0,011	0,001	0,014	0,000	0,007	0,000			
	MXL	24	0,117	0,080	0,149	0,141	0,144	0,144	0,136	0,035	0,072	0,058	0,046	0,047	0,042	0,041	0,030	0,040	0,038	0,041	0,017	0,050	0,016	0,034	0,014	0,000		
	PEL	25	0,227	0,187	0,264	0,255	0,255	0,257	0,251	0,100	0,154	0,135	0,128	0,126	0,079	0,061	0,062	0,066	0,078	0,124	0,089	0,134	0,074	0,104	0,073	0,025	0,000	
	PUR	26	0,067	0,041	0,096	0,098	0,103	0,102	0,088	0,006	0,022	0,014	0,005	0,007	0,082	0,093	0,070	0,086	0,074	0,010	-0,001	0,013	0,005	0,010	-0,001	0,016	0,087	0,000

Supplementary Figure 1. Pairwise FST analysis of human sub-populations – significance indicator

			African							European					East Asian					South Asian					American			
			ACB	ASW	ESN	GWD	LWK	MSL	YRI	CEU	FIN	GBR	IBS	TSI	CDX	CHB	CHS	JPT	KHV	BEB	GIH	ITU	PJL	STU	CLM	MXL	PEL	PUR
			**1**	**2**	**3**	**4**	**5**	**6**	**7**	**8**	**9**	**10**	**11**	**12**	**13**	**14**	**15**	**16**	**17**	**18**	**19**	**20**	**21**	**22**	**23**	**24**	**25**	**26**
**Afican**	ACB	**1**		-	-	-	-	-	-	+	+	+	+	+	+	+	+	+	+	+	+	+	+	+	+	+	+	+
	ASW	**2**	-		-	-	-	-	-	+	+	+	+	+	+	+	+	+	+	+	+	+	+	+	+	+	+	+
	ESN	**3**	-	-		-	-	-	-	+	+	+	+	+	+	+	+	+	+	+	+	+	+	+	+	+	+	+
	GWD	**4**	-	-	-		-	-	-	+	+	+	+	+	+	+	+	+	+	+	+	+	+	+	+	+	+	+
	LWK	**5**	-	-	-	-		-	-	+	+	+	+	+	+	+	+	+	+	+	+	+	+	+	+	+	+	+
	MSL	**6**	-	-	-	-	-		-	+	+	+	+	+	+	+	+	+	+	+	+	+	+	+	+	+	+	+
	YRI	**7**	-	-	-	-	-	-		+	+	+	+	+	+	+	+	+	+	+	+	+	+	+	+	+	+	+
**European**	CEU	**8**	+	+	+	+	+	+	+		-	-	-	-	+	+	+	+	+	+	-	+	-	+	-	+	+	-
	FIN	**9**	+	+	+	+	+	+	+	-		-	-	-	+	+	+	+	+	+	+	+	+	+	+	+	+	+
	GBR	**10**	+	+	+	+	+	+	+	-	-		-	-	+	+	+	+	+	+	+	+	+	+	-	+	+	+
	IBS	**11**	+	+	+	+	+	+	+	-	-	-		-	+	+	+	+	+	-	-	-	+	-	-	+	+	-
	TSI	**12**	+	+	+	+	+	+	+	-	-	-	-		+	+	+	+	+	+	+	+	+	+	-	+	+	-
**East Asian**	CDX	**13**	+	+	+	+	+	+	+	+	+	+	+	+		-	-	-	-	+	+	+	+	+	+	+	+	+
	CHB	**14**	+	+	+	+	+	+	+	+	+	+	+	+	-		-	-	-	+	+	+	+	+	+	+	+	+
	CHS	**15**	+	+	+	+	+	+	+	+	+	+	+	+	-	-		-	-	+	+	+	+	+	+	+	+	+
	JPT	**16**	+	+	+	+	+	+	+	+	+	+	+	+	-	-	-		-	+	+	+	+	+	+	+	+	+
	KHV	**17**	+	+	+	+	+	+	+	+	+	+	+	+	-	-	-	-		+	+	+	+	+	+	+	+	+
**South Asian**	BEB	**18**	+	+	+	+	+	+	+	+	+	+	-	+	+	+	+	+	+		-	-	-	-	-	+	+	-
	GIH	**19**	+	+	+	+	+	+	+	-	+	+	-	+	+	+	+	+	+	-		-	-	-	-	+	+	-
	ITU	**20**	+	+	+	+	+	+	+	+	+	+	-	+	+	+	+	+	+	-	-		-	-	+	+	+	+
	PJL	**21**	+	+	+	+	+	+	+	-	+	+	+	+	+	+	+	+	+	-	-	-		-	-	-	+	-
	STU	**22**	+	+	+	+	+	+	+	+	+	+	-	+	+	+	+	+	+	-	-	-	-		-	+	+	-
**American**	CLM	**23**	+	+	+	+	+	+	+	-	+	-	-	-	+	+	+	+	+	-	-	+	-	-		-	+	-
	MXL	**24**	+	+	+	+	+	+	+	+	+	+	+	+	+	+	+	+	+	+	+	+	-	+	-		+	+
	PEL	**25**	+	+	+	+	+	+	+	+	+	+	+	+	+	+	+	+	+	+	+	+	+	+	+	+		+
	PUR	**26**	+	+	+	+	+	+	+	-	+	+	-	-	+	+	+	+	+	-	-	+	-	-	-	+	+	

plus - p <0,05, minus - p >0,05

Supplementary Figure 1. Pairwise FST analysis of human sub-populations – p-values

			African						
			ACB	ASW	ESN	GWD	LWK	MSL	YRI
			1	2	3	4	5	6	7
Afican	ACB	1	*						
	ASW	2	0.45253+-0.0042	*					
	ESN	3	0.41125+-0.0046	0.14464+-0.0035	*				
	GWD	4	0.10009+-0.0028	0.12217+-0.0029	0.22582+-0.0038	*			
	LWK	5	0.15474+-0.0039	0.11415+-0.0032	0.34640+-0.0046	0.55183+-0.0052	*		
	MSL	6	0.13306+-0.0036	0.11039+-0.0031	0.32700+-0.0049	0.76854+-0.0044	0.83259+-0.0034	*	
	YRI	7	0.32531+-0.0041	0.26601+-0.0043	0.55529+-0.0051	0.58064+-0.0048	0.42402+-0.0047	0.50876+-0.0044	*
European	CEU	8	0.00000+-0.0000	0.00000+-0.0000	0.00000+-0.0000	0.00000+-0.0000	0.00000+-0.0000	0.00000+-0.0000	0.00000+-0.0000
	FIN	9	0.00000+-0.0000	0.00000+-0.0000	0.00000+-0.0000	0.00000+-0.0000	0.00000+-0.0000	0.00000+-0.0000	0.00000+-0.0000
	GBR	10	0.00000+-0.0000	0.00000+-0.0000	0.00000+-0.0000	0.00000+-0.0000	0.00000+-0.0000	0.00000+-0.0000	0.00000+-0.0000
	IBS	11	0.00000+-0.0000	0.00000+-0.0000	0.00000+-0.0000	0.00000+-0.0000	0.00000+-0.0000	0.00000+-0.0000	0.00000+-0.0000
	TSI	12	0.00000+-0.0000	0.00000+-0.0000	0.00000+-0.0000	0.00000+-0.0000	0.00000+-0.0000	0.00000+-0.0000	0.00000+-0.0000
East Asian	CDX	13	0.00000+-0.0000	0.00000+-0.0000	0.00000+-0.0000	0.00000+-0.0000	0.00000+-0.0000	0.00000+-0.0000	0.00000+-0.0000
	CHB	14	0.00000+-0.0000	0.00000+-0.0000	0.00000+-0.0000	0.00000+-0.0000	0.00000+-0.0000	0.00000+-0.0000	0.00000+-0.0000
	CHS	15	0.00000+-0.0000	0.00000+-0.0000	0.00000+-0.0000	0.00000+-0.0000	0.00000+-0.0000	0.00000+-0.0000	0.00000+-0.0000
	JPT	16	0.00000+-0.0000	0.00000+-0.0000	0.00000+-0.0000	0.00000+-0.0000	0.00000+-0.0000	0.00000+-0.0000	0.00000+-0.0000
	KHV	17	0.00000+-0.0000	0.00000+-0.0000	0.00000+-0.0000	0.00000+-0.0000	0.00000+-0.0000	0.00000+-0.0000	0.00000+-0.0000
South Asian	BEB	18	0.00000+-0.0000	0.00416+-0.0007	0.00000+-0.0000	0.00000+-0.0000	0.00000+-0.0000	0.00000+-0.0000	0.00000+-0.0000
	GIH	19	0.00000+-0.0000	0.00277+-0.0005	0.00000+-0.0000	0.00000+-0.0000	0.00000+-0.0000	0.00000+-0.0000	0.00000+-0.0000
	ITU	20	0.00000+-0.0000	0.00059+-0.0002	0.00000+-0.0000	0.00000+-0.0000	0.00000+-0.0000	0.00000+-0.0000	0.00000+-0.0000
	PJL	21	0.00000+-0.0000	0.00020+-0.0001	0.00000+-0.0000	0.00000+-0.0000	0.00000+-0.0000	0.00000+-0.0000	0.00000+-0.0000
	STU	22	0.00000+-0.0000	0.00079+-0.0003	0.00000+-0.0000	0.00000+-0.0000	0.00000+-0.0000	0.00000+-0.0000	0.00000+-0.0000
American	CLM	23	0.00000+-0.0000	0.00040+-0.0002	0.00000+-0.0000	0.00000+-0.0000	0.00000+-0.0000	0.00000+-0.0000	0.00000+-0.0000
	MXL	24	0.00000+-0.0000	0.00030+-0.0002	0.00000+-0.0000	0.00000+-0.0000	0.00000+-0.0000	0.00000+-0.0000	0.00000+-0.0000
	PEL	25	0.00000+-0.0000	0.00000+-0.0000	0.00000+-0.0000	0.00000+-0.0000	0.00000+-0.0000	0.00000+-0.0000	0.00000+-0.0000
	PUR	26	0.00000+-0.0000	0.00158+-0.0004	0.00000+-0.0000	0.00000+-0.0000	0.00000+-0.0000	0.00000+-0.0000	0.00000+-0.0000

			European					East Asian				
			CEU	FIN	GBR	IBS	TSI	CDX	CHB	CHS	JPT	KHV
			8	9	10	11	12	13	14	15	16	17
European	CEU	8	*									
	FIN	9	0.14979+-0.0041	*								
	GBR	10	0.43273+-0.0051	0.54302+-0.0047	*							
	IBS	11	0.33729+-0.0039	0.25245+-0.0047	0.49193+-0.0050	*						
	TSI	12	0.44174+-0.0050	0.22047+-0.0045	0.62667+-0.0054	0.79962+-0.0037	*					
East Asian	CDX	13	0.00000+-0.0000	0.00000+-0.0000	0.00000+-0.0000	0.00000+-0.0000	0.00000+-0.0000	*				
	CHB	14	0.00000+-0.0000	0.00000+-0.0000	0.00000+-0.0000	0.00000+-0.0000	0.00000+-0.0000	0.51242+-0.0043	*			
	CHS	15	0.00000+-0.0000	0.00000+-0.0000	0.00000+-0.0000	0.00000+-0.0000	0.00000+-0.0000	0.67399+-0.0045	0.43639+-0.0058	*		
	JPT	16	0.00000+-0.0000	0.00000+-0.0000	0.00000+-0.0000	0.00000+-0.0000	0.00000+-0.0000	0.75339+-0.0039	0.84596+-0.0039	0.70755+-0.0047	*	
	KHV	17	0.00000+-0.0000	0.00000+-0.0000	0.00000+-0.0000	0.00000+-0.0000	0.00000+-0.0000	0.91535+-0.0025	0.34046+-0.0042	0.74626+-0.0037	0.61152+-0.0053	*
South Asian	BEB	18	0.02683+-0.0015	0.03039+-0.0017	0.02247+-0.0016	0.06643+-0.0021	0.02247+-0.0014	0.00000+-0.0000	0.00000+-0.0000	0.00000+-0.0000	0.00000+-0.0000	0.00000+-0.0000
	GIH	19	0.05495+-0.0022	0.01000+-0.0009	0.01960+-0.0013	0.08356+-0.0027	0.03336+-0.0018	0.00000+-0.0000	0.00000+-0.0000	0.00000+-0.0000	0.00000+-0.0000	0.00000+-0.0000
	ITU	20	0.01643+-0.0014	0.03693+-0.0020	0.01851+-0.0012	0.05653+-0.0023	0.01455+-0.0013	0.00000+-0.0000	0.00000+-0.0000	0.00000+-0.0000	0.00000+-0.0000	0.00000+-0.0000
	PJL	21	0.09692+-0.0025	0.01426+-0.0011	0.02505+-0.0015	0.04861+-0.0019	0.03010+-0.0015	0.00000+-0.0000	0.00010+-0.0001	0.00010+-0.0001	0.00000+-0.0000	0.00000+-0.0000
	STU	22	0.04742+-0.0021	0.02812+-0.0016	0.02732+-0.0015	0.05356+-0.0020	0.02257+-0.0015	0.00000+-0.0000	0.00000+-0.0000	0.00010+-0.0001	0.00000+-0.0000	0.00000+-0.0000
American	CLM	23	0.36204+-0.0045	0.02921+-0.0017	0.09870+-0.0030	0.16612+-0.0038	0.13949+-0.0033	0.00000+-0.0000	0.00000+-0.0000	0.00000+-0.0000	0.00000+-0.0000	0.00000+-0.0000
	MXL	24	0.00703+-0.0008	0.00020+-0.0001	0.00119+-0.0004	0.00218+-0.0004	0.00158+-0.0004	0.00168+-0.0004	0.00238+-0.0005	0.00564+-0.0007	0.00327+-0.0007	0.00188+-0.0004
	PEL	25	0.00000+-0.0000	0.00000+-0.0000	0.00000+-0.0000	0.00000+-0.0000	0.00000+-0.0000	0.00000+-0.0000	0.00010+-0.0001	0.00010+-0.0001	0.00000+-0.0000	0.00000+-0.0000
	PUR	26	0.10078+-0.0031	0.00752+-0.0009	0.03208+-0.0019	0.14603+-0.0037	0.08148+-0.0027	0.00000+-0.0000	0.00000+-0.0000	0.00000+-0.0000	0.00000+-0.0000	0.00000+-0.0000

			South Asian					American			
			BEB	GIH	ITU	PJL	STU	CLM	MXL	PEL	PUR
			18	19	20	21	22	23	24	25	26
South Asian	BEB	18	*								
	GIH	19	0.29453+-0.0046	*							
	ITU	20	0.91456+-0.0025	0.15187+-0.0035	*						
	PJL	21	0.23255+-0.0042	0.39699+-0.0052	0.12058+-0.0035	*					
	STU	22	0.70934+-0.0043	0.28205+-0.0042	0.51173+-0.0052	0.49421+-0.0044	*				
American	CLM	23	0.07148+-0.0026	0.27463+-0.0041	0.03614+-0.0018	0.37135+-0.0044	0.12385+-0.0032	*			
	MXL	24	0.00574+-0.0008	0.03831+-0.0019	0.00059+-0.0002	0.05920+-0.0027	0.00861+-0.0009	0.06534+-0.0024	*		
	PEL	25	0.00000+-0.0000	0.00000+-0.0000	0.00000+-0.0000	0.00000+-0.0000	0.00000+-0.0000	0.00010+-0.0001	0.01881+-0.0013	*	
	PUR	26	0.06475+-0.0026	0.38838+-0.0040	0.02515+-0.0014	0.14632+-0.0037	0.06257+-0.0025	0.41481+-0.0047	0.04109+-0.0018	0.00000+-0.0000	*

Supplementary Figure 2. Genotyping of 1000 Genomes Project samples with unknown diplotypes.

	Our result						1000 Genomes						
**Sample**	**-52**	**-69**	**-513**	**-567**	**-631**	**-1142**	**-52**	**-69**	**-513**	**-567**	**-631**	**-1142**	**Diplotype**
HG00437	G	C	A	T	T	C	A	T	A	T	T	C	*A1
	A	T	G	G	G	G	A	T	G	G	G	G	*B1b
HG01384	G	C	A	T	T	C	A	T	A	T	T	C	*A1
	A	T	A	G	G	G	A	T	A	G	G	G	*B1a
HG02479	G	C	A	T	T	C	A	T	A	T	T	C	*A1
	A	T	A	G	G	C	A	T	A	G	G	C	*B2
HG03767	G	C	A	T	T	C	A	T	A	T	T	C	*A1
	A	T	A	G	G	G	A	T	A	G	G	G	*B1a
NA19064	G	C	A	T	T	C	A	T	A	T	T	C	*A1
	A	T	G	G	G	G	A	T	G	G	G	G	*B1b
NA19916	G	C	A	T	G	C	G	C	A	T	G	C	*A2
	A	T	A	G	G	C	G	C	A	G	G	C	*B2
HG03127	G	C	A	T	G	C	G	T	A	T	G	C	*A2
	A	T	A	G	G	C	A	T	A	G	G	C	*B2
NA18910	G	C	A	T	G	C	G	T	A	T	G	C	*A2
	A	T	A	G	G	C	A	T	A	G	G	C	*B2

Supplementary Figure 3. Comparison of LRA expression in equimolar plasmid mixtures.

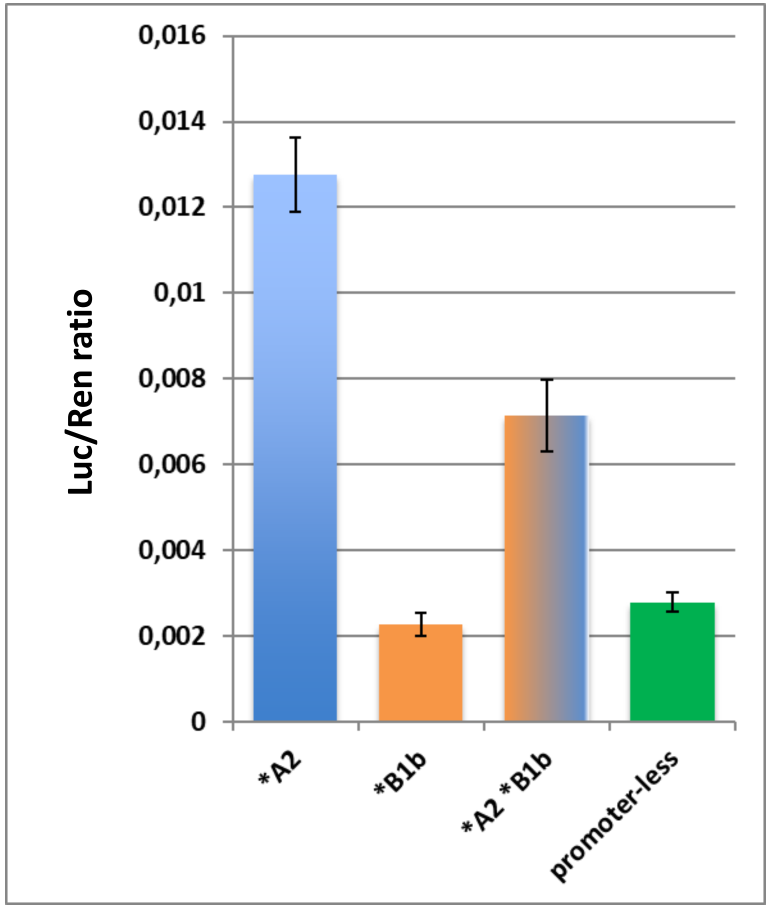

Supplementary Figure 4. TOPO PCR cloning for exact resolution of ambiguous haplotypes.

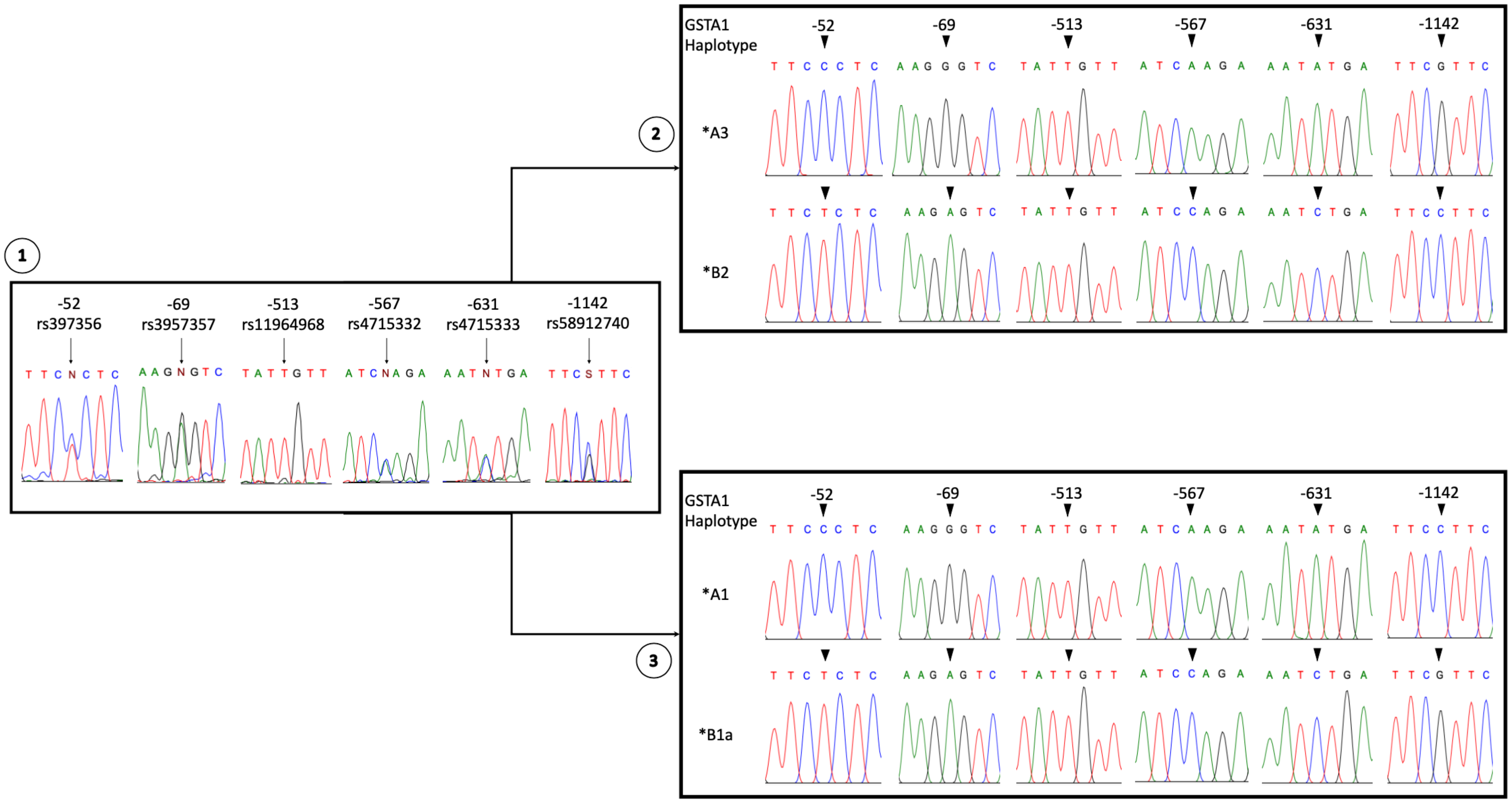

Supplementary Figure 5. Transcription factors binding at a specific position.

Position	pwmname	Allel	Score	Allel	Score
-52 / -69	SIX1	GC	7,80	AT	6,32
	RARA::RXRG	GC	13,40	AT	
	E2F6	GC	12,22	AT	
	SP1	GC	11,21	AT	
	NR2F1	GC	10,96	AT	
	Pparg::Rxra	GC	10,09	AT	
	KLF5	GC	10,06	AT	
	NR2F2	GC	9,33	AT	
	TFDP1	GC	8,59	AT	
	SP2	GC	8,57	AT	
	ZNF740	GC	8,49	AT	
	NR4A2	GC	8,19	AT	
	Nr2e1	GC	7,88	AT	
	SP3	GC	7,45	AT	
	RARA::RXRA	GC	7,44	AT	
	ZNF263	GC	7,11	AT	
	KLF16	GC	6,64	AT	
	ZBTB7C	GC	6,31	AT	
	MZF1(var.2)	GC	6,23	AT	
	SP8	GC	6,22	AT	
	RORA	GC	6,18	AT	
	EGR4	GC	6,16	AT	
	VDR	GC	6,13	AT	
	Nr2e1	GC		AT	11,79
	RARA::RXRG	GC		AT	7,22
	SIX2	GC		AT	6,88
	Pparg::Rxra	GC		AT	6,87
	ZBTB7C	GC		AT	6,78
	Pax2	GC		AT	6,51

Position	pwmname	Allel	Score	Allel	Score
-567	GATA1::TAL1	T	10,76	G	16,21
	FOSL1::JUNB	T	6,87	G	7,15
	FOSL1::JUN	T	6,17	G	6,23
	FOSL2::JUN	T	6,01	G	6,14
	MAFF	T	7,58	G	
	Nr2e1	T	6,81	G	
	Nkx2-5	T	6,81	G	
	MAFK	T	6,63	G	
	Gfi1	T	6,49	G	
	Six3	T	6,39	G	
	RHOXF1	T		G	7,88
	PITX3	T		G	7,36
	HSF4	T		G	6,68
	Pitx1	T		G	6,61
	ZNF354C	T		G	6,23
	HNF4G	T		G	6,12

Position	pwmname	Allel	Score	Allel	Score
-631	PRDM1	G	11,25	T	10,18
	IRF1	G	10,79	T	11,51
	IRF2	G	9,63	T	6,17
	SOX15	G	7,88	T	7,02
	SOX10	G	6,39	T	6,12
	Gfi1	G		T	8,83
	SIX1	G		T	7,54
	TCF7L2	G	9,00	T	
	Arid5a	G	7,67	T	
	LEF1	G	7,48	T	
	FOXD2	G	7,02	T	
	FOXC1	G	6,83	T	
	FOXL1	G	6,68	T	
	FOXC1	G	6,57	T	
	Tcf7	G	6,51	T	
	Dlx1	G	6,47	T	
	FOXP3	G	6,37	T	
	IRF7	G	6,30	T	
	Arid5a	G	6,14	T	
	FOXA1	G	6,06	T	

Position	pwmname	Allel	score	Allel	score
-1142	NR1A4::RXRA	G	6,602	C	
	NFIC::TLX1	G	6,756	C	

Supplementary Figure 5. Transcription factors binding at a specific position.

Position	pwmname	Allel	Score	Allel	Score
-513	CDX1	G	6,08	A	10,80
	HOXA13	G	6,21	A	10,96
	Hmx3	G	6,30	A	8,68
	Dlx1	G	6,54	A	6,76
	Hmx2	G	6,73	A	9,22
	ONECUT1	G	6,81	A	6,05
	RAX	G	6,83	A	6,58
	FOXK2	G	6,85	A	7,24
	FOXC1	G	6,86	A	8,57
	FOXF2	G	7,10	A	14,18
	FOXP3	G	7,28	A	10,82
	HOXA10	G	7,29	A	10,33
	FOXD2	G	7,67	A	10,73
	Hoxd8	G	7,68	A	12,18
	Foxd3	G	7,75	A	11,08
	Hoxd8	G	8,00	A	9,58
	BARHL2	G	8,11	A	7,71
	Hoxd9	G	8,22	A	10,64
	FOXC2	G	8,68	A	10,54
	GATA6	G	8,77	A	9,27
	CUX2	G	9,37	A	8,16
	FOXF2	G	9,39	A	8,03
	ONECUT2	G	9,42	A	8,69
	GATA2	G	9,52	A	10,18
	Gata1	G	9,58	A	10,65
	ONECUT3	G	9,70	A	10,59
	Foxj3	G	11,45	A	11,54
	BARHL2	G	11,87	A	8,84
-513	Foxj3	G		A	14,19
	Foxj2	G		A	13,58
	FOXG1	G		A	13,54
	FOXL1	G		A	12,01
	FOXP2	G		A	11,98
	Sox5	G		A	11,92
	SOX15	G		A	11,89
	SRY	G		A	11,79
	Foxq1	G		A	11,67
	CDX2	G		A	11,45
	FOXK2	G		A	11,21
	Hoxc9	G		A	11,20
	FOXO4	G		A	11,12
	FOXO6	G		A	11,06
	Hoxa9	G		A	10,77
	FOXC2	G		A	10,77
	Foxq1	G		A	10,69
	FOXK1	G		A	10,29
	FOXI1	G		A	10,25
	FOXO3	G		A	10,21
	SOX9	G		A	10,18
	Foxo1	G		A	10,00
	HOXB13	G		A	9,95
	FOXP1	G		A	9,92
	FOXD1	G		A	9,73
	Sox17	G		A	9,67
	SOX13	G		A	9,38
	ONECUT3	G		A	9,37
	Hmx1	G		A	8,69
-513	Hoxd8	G		A	8,63
	POU4F1	G		A	8,55
	ONECUT2	G		A	8,33
	HOXD13	G		A	8,26
	MNX1	G		A	8,22
	Sox6	G		A	8,06
	LMX1B	G		A	8,05
	FOXC1	G		A	7,95
	Foxa2	G		A	7,60
	Hoxd8	G		A	7,51
	FOXB1	G		A	7,31
	POU6F1	G		A	7,14
	POU6F1	G		A	7,14
	POU4F3	G		A	7,12
	Hoxd8	G		A	6,94
	ZNF384	G		A	6,82
	NKX6-2	G		A	6,79
	POU4F3	G		A	6,72
	FOXA1	G		A	6,66
	ESX1	G		A	6,53
	ONECUT1	G		A	6,51
	Prrx2	G		A	6,46
	POU4F1	G		A	6,43
	POU4F1	G		A	6,42
	Foxd3	G		A	6,33
	Arid5a	G		A	6,26
	Nobox	G		A	6,14
	FOXC2	G		A	6,14
	Arid3b	G		A	6,12
-513	Barhl1	G	6,88	A	
	Barhl1	G	6,69	A	
	GATA5	G	6,65	A	
	CUX1	G	6,54	A	
	BARHL2	G	6,52	A	
	GATA3	G	6,50	A	
	Hoxa11	G	6,37	A	
	Dlx1	G	6,32	A	
